# Effects of Temporal Characteristics on Pilots Perceiving Audiovisual Warning Signals Under Different Perceptual Loads

**DOI:** 10.3389/fpsyg.2022.808150

**Published:** 2022-02-10

**Authors:** Xing Peng, Hao Jiang, Jiazhong Yang, Rong Shi, Junyi Feng, Yaowei Liang

**Affiliations:** ^1^Institute of Aviation Human Factors and Cognitive Neuroscience, College of Flight Technology, Civil Aviation Flight University of China, Guanghan, China; ^2^Technical Support Center, Operation Control Department, Beijing Capital Airlines, Beijing, China; ^3^Flying Department of Southwest Branch, Air China Limited, Chengdu, China

**Keywords:** temporal characteristics, pilots, warning signals, audiovisual integration, perceptual load

## Abstract

Our research aimed to investigate the effectiveness of auditory, visual, and audiovisual warning signals for capturing the attention of the pilot, and how stimulus onset asynchronies (SOA) in audiovisual stimuli affect pilots perceiving the bimodal warning signals under different perceptual load conditions. In experiment 1 of the low perceptual load condition, participants discriminated the location (right vs. left) of visual targets preceded by five different types of warning signals. In experiment 2 of high perceptual load, participants completed the location task identical to a low load condition and a digit detection task in a rapid serial visual presentation (RSVP) stream. The main effect of warning signals in two experiments showed that visual and auditory cues presented simultaneously (AV) could effectively and efficiently arouse the attention of the pilots in high and low load conditions. Specifically, auditory (A), AV, and visual preceding auditory stimulus by 100 ms (VA100) increased the spatial orientation to a valid position in low load conditions. With the increase in visual perceptual load, auditory preceding the visual stimulus by 100 ms (AV100) and A warning signals had stronger spatial orientation. The results are expected to theoretically support the optimization design of the cockpit display interface, contributing to immediate flight crew awareness.

## Introduction

Pilots make use of information from the inner cockpit and outside the aircraft. About 80% of the information comes from the visual modality. Thus, pilots often suffer from visual signal overload, which may lead to flight accidents or dangerous situations. Audible information has been employed in the design of the cockpit to cope with visual overload, especially in the domain of warning signals. For example, Federal Aviation Regulation §25.1322 has been revised by Amendment No.131. It stipulates that warnings and caution alerts in the cockpit should provide prompt cues through no less than two distinct senses.

Existing studies have shown that the human brain can integrate visual and auditory signals into a unified, coherent, and meaningful perceptual content. This process can be called audiovisual integration (AVI). As indicated from the behavioral performances of the AVI, bimodal signals elicit faster and more accurate responses than unimodal (visual, auditory) signals alone (Hershenson, 1962; Miller, 1982; Frassinetti et al., 2002). Recently, the advantages of AVI have achieved an extensive application for designing warning signals, e.g., ground proximity warning systems and take-over request systems in autopilot. These warnings consist of multiple sensory signals, resulting in immediate awareness of the driver (Fitch et al., 2011; Prewett et al., 2011; Haas and Erp, 2014). This has suggested that bimodal signals capture spatial attention more effectively than that of unimodal signals (Santangelo et al., 2008).

The spatial cueing task was widely adopted to determine the ability of bimodal stimuli to capture attention (Posner, 1980). This task used an abrupt peripheral onset as exogenous cues to be presented on the left or right peripheral position. The target showed up at the identical (valid condition) or at the opposed location (invalid condition) to the previous cue. The reaction time to the target under the identical position was significantly faster than that under the opposed position, which is termed as the “spatial cueing effect.” Using this task, studies showed a spatial cueing effect of unimodal and bimodal cues with a comparable magnitude under the no-load condition. Also, only bimodal cues trigger a significant spatial cueing performance in the high perceptual load condition of rapid serial visual presentation (RSVP) task (Santangelo and Spence, 2007; Santangelo et al., 2008). Similar results were achieved in other high load tasks, e.g., visual search task and temporal order judgment task (Matusz and Eimer, 2011; Barrett and Katrin, 2012). These studies demonstrated that bimodal cues could elicit a larger spatial cueing effect than unimodal cues in high-load conditions. Importantly, the perceptual load modulates the spatial cueing effect elicited by bimodal stimuli. During the different flight phases (departure, cruise, and arrival), the perceptual load faced by pilots was changing. Thus, the spatial cueing effect of auditory, visual, and audiovisual (bimodal) warning signals under different perceptual load conditions should further be examined.

A vital factor that facilitates AVI is close temporal proximity, known as the temporal rule. The temporal binding window (TBW) reflects the range of stimulus onset asynchronies (SOA) with audiovisual stimuli integrated for forming a single percept. Studies found that when audiovisual stimuli are presented at approximately the same time, the effect of AVI is pronounced (Stevenson et al., 2012). However, some found that audiovisual stimuli are presented asynchronously, for instance, when SOA was 40 ms (Lewald and Getzmann, 2011), 100 ms (Van de Par and Kohlrausch, 2000), and even 250 ms (Mcdonald et al., 2000), produced the significant AVI effect. For integrating of the simple stimuli (e.g., visual flashes and auditory beeps), the TBW is approximately 20–80 ms (Keetels and Vroomen, 2005). In terms of more complex and meaningful stimuli (e.g., audiovisual speech), the TBW increases to a few hundred milliseconds (Vroomen and Keetels, 2010). Several studies found the differential width of the TBW based on different groups. For instance, as compared with controls, people with schizophrenia (Stevenson et al., 2017) and autism spectrum disorders (Stevenson et al., 2014) have wider TBWs. Musicians relative to non-musicians showed significantly narrower TBWs for music and sine-wave speech (Lee and Noppeney, 2014). As impacted by the differential life-related perceptual experiences, a simple sensory training can change the TBW and may impact multisensory processing (Donohue et al., 2010). It is hypothesized that whether the prior experiences and life history of the special group of long-term training pilots might affect their multisensory processing. Besides, the width of the TBW was also strongly dependent on task difficulty related to perceptual load and tasks with higher perceptual load produced wider TBWs (Stevenson and Wallace, 2013).

In summary, perceptual load and SOA in audiovisual stimuli affected the AVI of bimodal warning signals. The present study examines how temporal characteristics affect pilots perceiving bimodal warning signals under different perceptual loads. We used the spatial cueing task and manipulated the types of warning signal and cue validities to investigate the spatial cueing effect of auditory, visual, and audiovisual (bimodal) warning signals, and how temporal characteristics affect the pilots who are perceiving a bimodal warning under different perceptual loads. Participants were required to conduct the discrimination of the position (right vs. left) of visual targets preceded by these different warning signals in experiment 1 (low perceptual load). In experiment 2 (high perceptual load), subjects were to perform an additional demanding central RSVP task, in which they were required to identify digit numbers in a series of presented letters. We hypothesized that: (1) bimodal warning signals were likely to have significantly greater effectiveness than unimodal warning signals to capture the attention of a pilot and (2) the perceptual load modulates the effects of temporal characteristics for bimodal warning signals.

## Experiment 1

### Method

#### Participants

Twenty-four pilots (average age: 22.08 ± 1.06 years; age scope: 20–24 years) were recruited from the Civil Aviation Flight University of China. All participants had normal or corrected-to-normal vision, were all right-handed, and did not have any neurological or psychiatric disorders history. They got aviation commercial licenses from the Civil Aviation Administration of China (CAAC) and have logged an average of more than 230 h of flight in simulators and real aircraft. This study was approved by the Ethics Committee of Civil Aviation Flight University of China and was performed in accordance with the approved guidelines and the Declaration of Helsinki. The written informed consent was offered by each subject engaged.

#### Apparatus and Materials

There existed five warning signals serving as the cue, presented at the left or right side of the screen to capture the attention of the participants. The visual (V) warning signal was a black rectangle (size:0.2° × 0.1°; 4.5° away from the center of the screen). The auditory (A) warning signal was pure tones of 1,000 Hz (65 dB, 100 ms, 10 ms rising and falling time) played through earphones. Unimodal V and A warning signals were presented for 100 ms. Moreover, visual and auditory signals were combined to generate bimodal warning cues. The visual signal in the bimodal cues could be presented simultaneously with, 100 ms prior to, or 100 ms after the auditory signal. These three temporal conditions were labeled as AV, VA100, or AV100, respectively. Thus, five warning signal cue types were V, A, AV, VA100, and AV100, respectively. In the target screen, a visual target was presented, i.e., a red (RGB: 255, 0, 0; 27.5 cd/m^2^) solid circle (size: 1° × 1°) presented at the identical (valid cue) or opposite (invalid cue) location to the previous cue.

**FIGURE 1:**
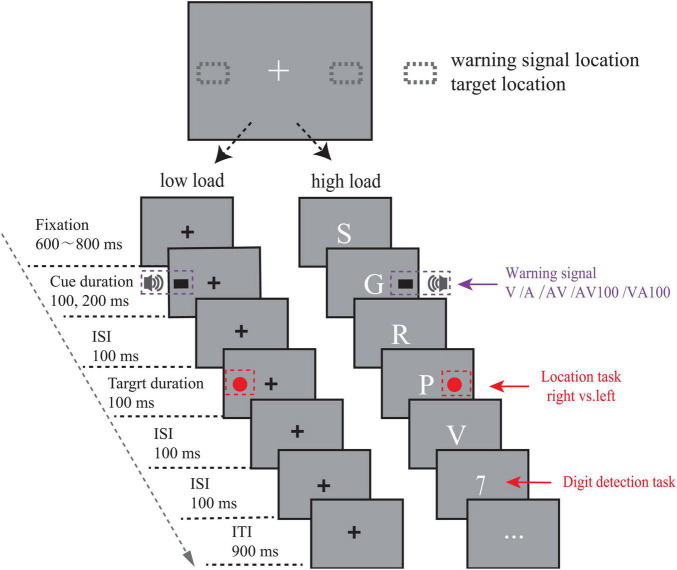
Illustration of the stimuli and experimental procedure. The locations of stimuli are shown in the above panel, and the procedure for a single trial of the two experiments is shown in the panel below. In experiment 1 (low load condition), a warning signal cue (the black rectangle with pure tones) was shown on the left side of the screen. A visual target (the red solid circle) was also shown on the left (i.e., valid cue condition). Participants were asked to respond to the location of the target as fast and accurately as possible. Warning signal cues (V/A/AV/AV100/VA100) represented visual, auditory, visual and auditory stimulus presented simultaneously, auditory preceding visual stimulus by 100 ms, visual preceding auditory stimulus by 100 ms, respectively. In Experiment 2 (high load condition), the procedure was the same as in experiment 1. The difference was the replacement of the center fixation “+” by a central stream of visual letters and target digits with the occasional presentation, which covered 17 letters (B, C, D, E, F, J, K, L, M, N, P, R, S, T, Y, X, and Z).

The experimental design was a 5 (warning signal cue type: V, A, AV, VA100, and AV100) × 2 (cue validity: 50% valid cue and 50% invalid cue) factorial design. As shown in Figure 1, the fixation stimulus had the presentation inside the display center and lasted for 600–800 ms. Then, one type of warning cues (V, A, AV, VA100, AV100) was randomly presented to left or right of the central fixation, lasting for 100 ms (V, A, AV) or 200 ms (VA100, AV100). After an interval time of 100 ms, a target stimulus (red solid circle) was randomly shown on the identical (valid cue location) or opposite (invalid cue location) sides of the warning signal, lasting for 100 ms duration. The next trial started 900 ms later.

Participants were tested in a quiet and dimly lit room. The stimuli were presented on a 24-in computer monitor with a refresh rate of 60 Hz. The distance between the eyes of the participants and the computer monitor was about 60 cm. The subjects participating in the experiment were instructed to respond as soon and as accurately as possible. When the target showed up on the left side of the screen, the “←” key on the keyboard should be pressed. Otherwise, the “→” key should be pressed. The experiment covered a total of 1,080 trials, arranged in 6 blocks of 180 trials within each block. The experiment lasted for approximately 40 min.

#### Data Analysis

Incorrect trials and trials with the reaction time below 100 ms or those over 1,000 ms were deleted and not calculated, because they were assumed to be the result of anticipation or not paying attention to the task, respectively. In addition to the reaction time and accuracy, we also calculated the inverse efficiency score (IES). This single index was calculated by combining RT and accuracy: RT/percentage of correct responses (Townsend and Ashby, 1983). Please note that the smaller the IES, the better the performance. The 5 × 2 repeated measures analysis of variance was used to analyze the accuracy, reaction time, and IES data with the factor warning signals (V, A, AV, VA100, and AV100) and cue validity (valid cue and invalid cue). The Bonferroni correction was used for multiple comparisons correction. The effect size of the partial eta-squared (η_p_^2^) was calculated for the ANOVA. All statistical levels were set to.05.

### Results

#### Accuracy

**FIGURE 2:**
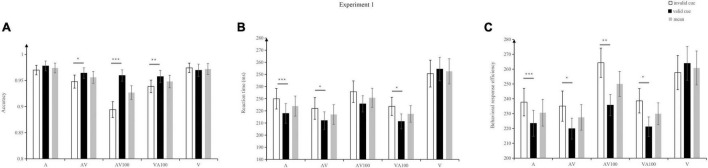
Mean accuracy **(A)**, reaction times **(B)**, and inverse efficiency score **(C)** under different warning signals and cue validities in Exp. 1. V/A/AV represented visual, auditory, and audiovisual warning signal cues, respectively. The number 100 represented the interval time between two stimuli. Error bars denote ± SE. ***p < 0.001, **p < 0.01, and *p < 0.05.

Figure 2A illustrated the results: the main effect of warning signals is significant, *F* (4, 92) = 22.55, *p* = 0.001, η_p_^2^ = 0.50. The accuracy of A (97.4%) was significantly higher than that of AV (95.6%, *p* = 0.007), AV100 (92.7%, *p* < 0.001), and VA100 (94.8%, *p* = 0.009); The accuracy of AV (95.6%) was significantly higher than AV100 (92.7%, *p* < *0.001*); The accuracy of V (97.2%) was significantly higher than AV (95.6%, *p* = 0.013), AV100 (92.7%, *p* < 0.001), and VA100 (94.8%, *p* = 0.019). The accuracy of VA100 (94.8%) was significantly higher than AV100 (92.7%, *p* = 0.023. The main effect of cue validity was significant, *F* (1, 23) = 55.24, *p* < 0.001, η_p_^2^ = 0.71. The accuracy of valid cues (96.6%) was significantly higher than the invalid cues (94.5%), *p* < 0.001. A significant interaction was identified between warning signals and cue validity, *F* (4, 92) = 15.77, *p* < 0.001, η_p_^2^ = 0.40. To be specific, accuracy to validly cued AV targets (*M* = 96.4%, *SD* = 4.95) was significantly higher than to invalidly cued AV targets (*M* = 94.8%, *SD* = 6.12), *t*(23) = 2.67, *p* = 0.014, and *d* = 0.28; Accuracy to validly cued AV100 targets (*M* = 96%, *SD* = 5.32) was significantly higher than to invalidly cued AV100 targets (*M* = 89.4%, *SD* = 7.48), *t*(23) = 6.97, and *p* < 0.001, and *d* = 0.99; Moreover, compared to invalidly cued VA100 targets (*M* = 93.9%, *SD* = 5.94), accuracy to validly cued VA100 targets (*M* = 95.7%, *SD* = 5.79) was significantly higher, *t*(23) = 6.97, *p* = 0.009, and *d* = 0.30. No difference in accuracy was identified between validly and invalidly cued A and V targets.

Three types of warning signals (AV, AV100, and VA100) can promote the detection of targets on the valid cue condition. Because the accuracy rates of the warning signals are above 95%, we further analyze the reaction time.

#### Reaction Time

Figure 2B presents the results: the main effect of warning signals is significant, *F* (4, 92) = 30.90, *p* < 0.001, η_p_^2^ = 0.573. The reaction time of A (224 ms) was significantly faster than that of V (253 ms, *p* < 0.001); The reaction time of AV (217 ms) was significantly faster than that of A (224 ms, *p* = 0.003), AV100 (231 ms, *p* < 0.001), and V(253 ms, *p* < 0.001); The reaction time of AV100 (231 ms) was significantly faster than that of V (253 ms, *p* = 0.004); The reaction time of VA100 (217 ms) was significantly faster than that of AV100 (231 ms, *p* = 0.001) and V(253 ms, *p* < 0.001). The main effect of cue validity was significant, *F* (1, 23) = 5.24, *p* = 0.032, η_p_^2^ = 0.19, the reaction time of valid cue (224 ms) was significantly faster than that of invalid cue (232 ms).

There was a significant interaction between warning signals and cue validity, *F* (4, 92) = 6.65, *p* < 0.001, η_p_^2^ = 0.22. To be specific, reaction time to validly cued AV targets (*M* = 212 ms, *SD* = 35 ms) was significantly faster than to invalidly cued AV targets (*M* = 222 ms, *SD* = 44 ms), *t*(23) = 2.28, *p* = 0.032, and *d* = 0.25; Reaction time to validly cued VA100 targets (*M* = 211 ms, *SD* = 30 ms) was significantly faster than to invalidly cued VA100 targets (*M* = 224 ms, *SD* = *37* ms), *t*(23) = 2.78, *p* = 0.011, and *d* = 0.38; Also, reaction time to validly cued A targets (*M* = 218 ms, *SD* = 40 ms) was significantly faster than to invalidly cued A targets (*M* = 230 ms, *SD* = 41 ms), *t*(23) = 4.51, *p* < 0.001, and *d* = 0.30; No difference was identified in reaction time between validly and invalidly cued V and AV100 targets. From the results of the reaction time, A, AV and VA100 warning signals produced a significant exogenous cueing effect, demonstrating that the mentioned three types of warning signals are capable of capturing the pilot’s attention effectively.

#### Inverse Efficiency Score

Figure 2C presents the results: the main effect of warning signals is significant, *F* (4, 92) = 23.189, *p* < 0.001, η_p_^2^ = 0.502. The IES of AV (227) was significantly smaller than that of AV100 (250, *p* < 0.001) and V (261, *p* < 0.001); The IES of VA100 (230) was significantly smaller than that of AV100 (250, *p* < 0.001) and V (261, *p* < 0.001); The IES of A (231) was significantly smaller than that of AV100 (250, *p* < 0.001) and V (261, *p* < 0.001). The main effect of cue validity was significant, *F* (1, 23) = 11.23, *p* = 0.003, η_p_^2^ = 0.328. The IES of valid cue (232) was significantly smaller than that of invalid cue (247).

There was a significant interaction between warning signals and cue validity, *F* (4, 92) = 10.028, *p* < 0.001, η_p_^2^ = 0.304. To be specific, IES to validly cued AV targets (*M* = 220, *SD* = 34) was significantly smaller than to invalidly cued AV targets (*M* = 235, *SD* = 50), *t*(23) = 2.51, *p* = 0.019, and *d* = 0.34; The IES to validly cued VA100 targets (*M* = 221, *SD* = 32) was significantly smaller than to invalidly cued VA100 targets (*M* = 239, *SD* = 40), *t*(23) = 3.14, *p* = 0.005, and *d* = 0.5; Also, IES to validly cued A targets (*M* = 223, *SD* = 43) was significantly smaller than to invalidly cued A targets(*M* = 238, *SD* = 45), *t*(23) = 4.52, *p* < 0.001, and *d* = 0.34; IES to validly cued AV100 targets (*M* = 236, *SD* = 35) were significantly smaller than to invalidly cued AV100 targets (*M* = 264, *SD* = 48), *t*(23) = 3.67, *p* = 0.001, and *d* = 0.65; No difference was identified in IES between validly and invalidly cued V targets.

The IES results showed AV, VA100, and A warning signals had better IES regardless of cue validity. Considering the cue validity condition, A, AV, AV100, and VA100 produced better IES to valid target stimuli.

## Experiment 2

The visual load faced by pilots will change in different flight stages. For example, during the taxiing and cruise phase, there involve low visual loads. During the take-off, climb and final approach phase, the pilot needs to pay more attention to monitor the flight parameters of the cockpit, which involves high visual loads. With the visual load increased, will the TBW between the V and A signals affect the AVI? For the mentioned reason, experiment 2 employed the RSVP stream paradigm to increase the visual load, examining which warning signal cues are more effective to capture the spatial attention and how temporal characteristics affect the bimodal warning signals under a high perceptual load.

### Methods

#### Participants

Twenty-four pilots (age range: 20–24 years; mean age: 22.09 ± 1.06 years) were recruited from the Civil Aviation Flight University of China. Two participants were excluded from further analyses because their accuracy rates were below 90% in the digit detection task of experiment 2. The written informed consent was provided by all subjects participating in the experiment. All pilot participants had normal or corrected-to-normal vision, were all right-handed, and did not have any neurological or psychiatric disorders history. They got aviation commercial licenses from the CAAC and have logged an average of more than 230 h of flight in simulators and real aircraft.

#### Apparatus and Materials

In the high-load condition, the center fixation in experiment 1 was replaced by a central stream of visual letters for target digits with occasional presentation, which is named RSVP stream. The set of distractors within the RSVP covered 17 letters (B, C, D, E, F, J, K, L, M, N, P, R, S, T, Y, X, and Z), which does not require a response, and the set of targets covered six digits (2, 3, 4, 5, 6, and 9; 1° × 1°). The five distractor letters in the stream were selected randomly before each trial with the sole restriction that no distractor was repeated within a given stream. Each letter was presented for 100 ms. The warning signal was presented as a cue in the third-stream position, and equiprobably on either side of fixation, while the target digit appeared equiprobably in the sixth or seventh stream position. When presented, the red circle target of the spatial cueing task showed up in the fifth position after the warning cue. The target digit had the presentation on 20% of the trials, whereas the red circle peripheral target had the presentation on the remaining 80% of the trials. Subjects participating in the experiment should execute the location task (requiring a discrimination response), which was identical to experiment 1, and the digit detection task (press the “↓” key after seeing the target digit in the screen center). The experiment lasted for approximately 40 min. There were 1,080 trials in total, which consisted of 864 trials of the location task and 216 trials of the digit detection task.

#### Data Analysis

The standard of data elimination and data analysis method of accuracy and reaction time were the same as experiment 1. In experiment 2, pilots participating in the experiment should complete both the location and the digit detection tasks. Two participants were excluded from further analyses because their accuracy rates were below 90% in the digit detection task. The purpose of the digit task was to increase the visual load, so we did not analyze the data from this task. The following analysis was only for the location task. The Bonferroni correction was used for multiple comparisons correction. The effect size of the partial eta-squared (η_p_^2^) was calculated for the ANOVA. All statistical levels were set to.05.

### Results

#### Accuracy

**FIGURE 3:**
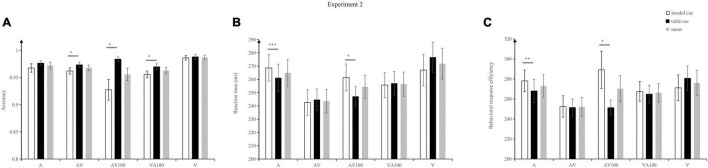
Mean accuracy **(A)** and reaction times **(B)**, and inverse efficiency score **(C)** under different warning signals and cue validities in Exp. 2. Error bars denote ± SE. ***p < 0.001, **p < 0.01, and *p < 0.05.

The results are shown in Figure 3A: the main effect of warning signals was observed, *F* (4, 84) = 4.58, *p* = 0.002, η_p_^2^ = 0.18. The accuracy of V (98.7%) was significantly higher than that of AV (96.8%, *p* = 0.02) and VA100 (96.3%, *p* = 0.004). The main effect of cue validity was significant, *F* (1, 21) = 13.19, *p* = 0.002, η_p_^2^ = 0.39. The accuracy of valid cues (97.9%) was significantly higher than that of invalid cues (96%), *p* = 0.002.

There was a significant interaction between cue validity and warning signals, *F* (4, 84) = 4.12, *p* = 0.004, η_p_^2^ = 0.16. To be specific, the accuracy to validly cued AV targets (*M* = 97.4%, *SD* = 2.17) were significantly higher than to invalidly cued AV targets (*M* = 96.2%, *SD* = 2.69), *t*(21) = 2.15, *p* = 0.04, and *d* = 0.49; The accuracy to validly cued AV100 targets (*M* = 98.4%, *SD* = 1.9) were significantly higher than to invalidly cued AV100 (*M* = 92.8%, *SD* = 10.23) targets, *t*(21) = 2.64, *p* = 0.015, and *d* = 0.59; the accuracy to validly cued VA100 targets (*M* = 97%, *SD* = 2.6) were significantly higher than to invalidly cued VA100 (*M* = 95.6%, *SD* = 2.96) targets, *t*(21) = 2.29, *p* = 0.014, and *d* = 0.5; There was no difference in accuracy between different validly conditions on A and V targets. Three types of warning signals (AV, AV100, and VA100) can promote the detection of targets on the valid cue condition.

#### Reaction Time

The results are presented in Figure 3B: a main effect of warning signals was observed, *F* (4, 84) = 21.38, *p* < 0.001, η_p_^2^ = 0.50. The reaction time of AV (244 ms) was significantly faster than that of V (272 ms, *p* < 0.001), A (265 ms, *p* < 0.001), AV100 (254 ms, *p* = 0.001), and VA100 (256 ms, *p* < 0.001). The reaction time of AV100 (254 ms) was significantly faster than that of V (272 ms, *p* = 0.01). The reaction time of VA100 (256 ms) was significantly faster than that of A (265 ms, *p* = 0.035) and V (272 ms, *p* = 0.004). The main effect of cue validity was not significant, *F* (1, 21) = 0.44, *p* = 0.51.

There was a significant interaction between cue type and cue validity, *F* (4, 84) = 6.81, *p* < 0.001, η_p_^2^ = 0.25. To be specific, reaction time to validly cued A targets (*M* = 261 ms, *SD* = 48 ms) were significantly faster than to invalidly cued A targets (*M* = 269 ms, *SD* = 46 ms), *t*(21) = 4.34, *p* < 0.001, and *d* = 0.17; Reaction time to validly cued AV100 targets (*M* = 247 ms, *SD* = 34 ms) significantly faster that to invalidly cued AV100 targets (*M* = 262 ms, *SD* = 48 ms), *t*(21) = 2.32, *p* = 0.03, and *d* = 0.35. There was no difference in reaction time between different validly conditions on AV, V, and VA100. From the results of the reaction time, A and AV100 warning signal exerted an significant spatial cueing effect.

#### Inverse Efficiency Score

Figure 3C presents the results: the main effect of warning signals was significant, *F* (4, 84) = 4.543, *p* = 0.002, η_p_^2^ = 0.178. The IES of AV (252) was significantly smaller than that of V (266, *p* < 0.001), A (273, *p* < 0.001), and VA100 (276, *p* < 0.001).

The main effect of cue validity was significant, *F* (1, 21) = 5.219, *p* = 0.033, η_p_^2^ = 0.199. The IES of valid cue (263) was significantly smaller than that of invalid cue (271). There was a significant interaction between warning signals and cue validity, *F* (4, 84) = 4.406, *p* = 0.003, η_p_^2^ = 0.173. To be specific, IES to validly cued A targets (*M* = 268, *SD* = 55) were significantly smaller than to invalidly cued A targets (*M* = 278, *SD* = 51), *t*(21) = 3.86, *p* = 0.001, and *d* = 0.19; Also, IES to validly cued AV100 targets (*M* = 251, *SD* = 36) were significantly smaller than to invalidly cued AV100 targets (*M* = 289, *SD* = 88), *t*(21) = 2.21, *p* = 0.039, and *d* = 0.5; No difference was identified in IES between validly and invalidly cued V, AV, and VA100 targets.

#### Comparison Between Low and High Perceptual Loads

**FIGURE 4:**
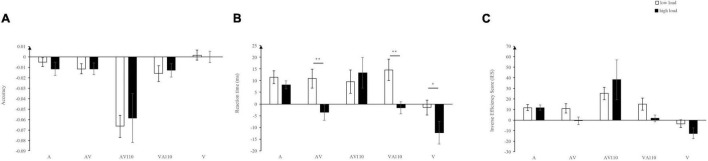
Validity effects in terms of accuracy **(A)**, reaction times **(B)**, and inverse efficiency score **(C)**, using data from the two experiments. Error bars denote ± SE. **p < 001, and *p < 0.05.

Finally, data from the two experiments were combined for further analysis. We focused on the validity effect, which refers to the difference of accuracy, reaction time, or IES between invalid-cue and valid-cue conditions. Then, we would like to investigate how the validity effect would be influenced by perceptual loads and types of warning signals. To this end, three 2 (perceptual load level) by 5 (types of warning signals) within-subjects ANOVAs were performed, for accuracy, reaction time, and IES, respectively. The results are shown in Figures 4A–C, respectively. When analyzing, data from the twenty shared subjects from the two experiments were used.

Results showed that, for accuracy, the main effect of warning signal cue type was significant, *F* (4, 76) = 11.05, *p* < 0.001, η_p_^2^ = 0.368. Validity effect (in terms of accuracy) for AV100 (−0.062) was smaller than A (−0.008, *p* = 0.016), AV (−0.012, *p* = 0.032) and V (0.001, *p* = 0.005). The main effect of perceptual load level was not significant, *F* (1, 19) = 0.1, *p* = 0.92, nor was the interaction between the two factors, *F* (4, 76) = 0.211, *p* = 0.931.

For reaction times, the main effect of warning signal cue type was significant, *F* (4, 76) = 8.366, *p* < 0.001, η_p_^2^ = 0.306. Validity effect of V (−6.88 ms) was significantly smaller than A (9.84 ms, *p* = 0.001), AV (3.65 ms, *p* = 0.006), AV100 (11.44 ms, *p* = 0.03), and VA100 (6.5 ms, *p* = 0.003). The main effect of perceptual load level was significant, *F* (1, 19) = 8.03, *p* = 0.011, η_p_^2^ = 0.297. Validity effect (in terms of reaction times) in low load condition (9 ms) was significantly larger than that in high load condition (0.82 ms, *p* = 0.011). Importantly, there was a significant interaction between cue type of warning signal and perceptual load level, *F* (4, 76) = 4.422 *p* = 0.003, η_p_^2^ = 0.189. Simple effect analysis revealed a significantly smaller validity effect in high load condition compared with low load condition for AV (*p* = 0.003), VA100 (*p* = 0.007), and V (*p* = 0.025). In low load condition, validity effect of V (−1.48 ms) was significantly smaller than A (11.43 ms, *p* = 0.03), AV (10.89 ms, *p* = 0.015), and AV100 (9.56 ms, *p* = 0.034). In high load condition, validity effect of A (8.26 ms) was significantly larger than AV (−3.59 ms, *p* = 0.014), VA100 (−1.60 ms *p* = 0.34), and V (−12.29 ms, *p* = 0.003). AV100 (13.31 ms) was significantly larger than V (−12.29 ms, *p* = 0.037).

For IES, it was similar with the accuracy. The main effect of warning signal cue type was significant, *F* (4, 76) = 7.722, *p* < 0.001, η_p_^2^ = 0.289. Validity effect (in terms of IES) of V (−8.09) was significantly smaller than A (11.76, *p* = 0.001), AV (5.12, *p* = 0.002), AV100 (31.64, *p* = 0.03), and VA100 (8.43, *p* = 0.001). The main effect of perceptual load level was not significant, *F* (1, 19) = 0.982, *p* = 0.334, nor was the interaction between the two factors, *F* (4, 76) = 1.379, *p* = 0.249.

## Discussion

### The Effectiveness of Different Types of Warning Signals

This study tested several types of unimodal and multimodal warning signals to explore their effects in capturing the attention of the pilot. In doing so, reaction times, accuracy, and IES were analyzed. For the sake of clarity, we discussed the IES results as it provides an integration of the other two. The main effect of warning signals for IES in two experiments showed that AV, VA100, and A warning signals produced better processing advantages in low load conditions, AV warning cues produced a better processing advantage in high load conditions.

In low load condition, the IES of AV, VA100, and A was significantly smaller than that of V and AV100. In other words, AV, VA100, and A warning signals showed advantages in arousal. Firstly, AV warnings had a better task performance. This result is consistent with previous studies: when audiovisual stimuli are presented at approximately the same time, the effect of AVI is pronounced (Stevenson et al., 2012). Secondly, unimodal A cues can be significantly more effective than V alone in enhancing the performance of the pilot, even better than bimodal cues (AV100). Auditory displays are more effective than visual displays alone in improving user performance and effectively attracting the attention of the drivers (Edworthy and Hellier, 2006; Haas and Edworthy, 2006). Santangelo et al. (2006, 2008) investigated whether multisensory cues might be more effective in capturing the spatial attention of a person than unimodal cues. Their results showed the reaction time of A cues was the fastest, significantly faster than AV and V, which is partly consistent with ours. When pilots were required to accomplish a visual-spatial information processing, their visual attention resources were occupied, so the auditory-led warning signals showed the dominance effect. Thirdly, some studies found that AVI is often the most pronounced when the stimuli from different modalities are presented asynchronously. Specifically, VA100 produced better IES than AV100 in the low load condition of our study. Previous studies showed bimodal signals in which visual preceding auditory stimulus by about 100 ms can produce the maximal response enhancement (Rowland et al., 2007; Perrault et al., 2011). This can be due to the differences in sensory conduction and processing time, and sensory input time may not be precisely aligned in time (King and Palmer, 1985). Auditory signals arrive in the brain about 30–50 ms earlier than visual signals, so the integration can probably occur when visual stimulation precedes auditory stimulation.

In the high load condition, the IES of AV was significantly smaller than that of V, A, and VA100. An AV warning had a better task performance than V or A warning alone regardless of other AV conditions (VA100). This result is consistent with our hypothesis and previous studies. Multimodal warnings can potentially enhance risk communication (van Erp et al., 2015). The bimodal stimulus presented synchronously elicited faster and more accurate responses than unimodal visual or auditory stimuli (Raab, 1962; Stein and Meredith, 1993). According to the temporal principle of audiovisual processing, the synchronous occurrence of the A and V stimuli of a multisensory stimulus can be a percept into a single and coherent perceptual representation (Meredith et al., 1987; Stein and Meredith, 1993; Senkowski et al., 2007). When V and A signals are presented simultaneously, like the AV warning signals in this study, they can elicit more rapid and more precise responses in contrast to the auditory or visual modality alone. This advantage can be termed as a “redundant signals effect” (Hershenson, 1962).

### Comparison Between Low and High Perceptual Loads

We compared the validity effect under different perceptual loads. There was a significant interaction between cue type of warning signal and perceptual load level in reaction time, but not in accuracy and IES. Specifically, there was a significant validity effect of reaction time (spatial cueing effect) for AV and VA100 in low load conditions, but no such effect in high load conditions. The perceptual load modulates the effects of temporal characteristics for bimodal warning signals. Previous studies showed the spatial cueing effect was found in bimodal (audio-tactile and audiovisual) cues both in high and low perceptual load, but no spatial cueing effect was found in unimodal auditory, visual, or tactile cues under conditions of high load (Santangelo and Spence, 2007; Santangelo et al., 2008; Ho et al., 2009). In other words, only bimodal cues still captured attention under conditions of high perceptual load. However, in our study, AV cues showed no processing advantage under high load condition. On the contrary, AV, VA100, and A elicited faster responses to the valid target stimuli under low load conditions. The AV100 and A produced faster response time to the valid target stimuli under high load conditions. The additional auditory signal can effectively attract the attention of the pilot under the visual overload situation, especially in A and AV100 conditions. There are some reasons for this result: as visual load increases, auditory stimulus plays an important role in warning. The task does not consume all available perceptual resources in low-load conditions, and pilots can process additional stimuli. However, high-load tasks consume considerable attention resources, causing additional task-related information to be less processed. As indicated from perceptual load theory (Lavie, 2005), attention is a limited capacity system that proceeds automatically until such a capacity is reached. Reduced attention capacity for processing multisensory stimuli can impair multisensory temporal processing. This implies that the perceptual operations under high load conditions take more time to reachcompletion, suggesting additional perceptual resources can be used to process a multisensory temporal in low load conditions. Flying requires pilots to continuously monitor various instrument information in the cockpit and the environmental information outside the cockpit *via* predominantly visual input. Around 80% of the information necessary for the pilots is acquired through the visual system (Dehais et al., 2017). We speculate with the visual overload in the RSVP task; it can be argued that presenting warning signals *via* the auditory modality causes less workload of pilots compared to presenting the same information *via* the visual modality. Therefore, pilots benefited most from auditory-led warning signals (A and AV100).

It should be noted that there was no replication of Posner’s classic facilitation effect under visual conditions. This is inconsistent with most previous studies, but there are a few studies having similar results with ours. For example, Van der Stoep et al. (2015) used an exogenous cue-target paradigm and modulated the modality of the target stimulus (A, V, and AV) to investigate the relationship between exogenous spatial attention and audiovisual integration. They found the validity effect (facilitating effect) appeared in the V and AV targets, but not for A targets in the location task (experiment 2). This result was like ours, when the cue and the target were from the same modality (our study: V cue_ V target; Van der Stoep et al: A cue_ A target), no validity effect was produced in this modality. Some researchers modulated the modality of the cue stimulus to investigate whether multisensory cues might be more effective in capturing the spatial attention of a person than unimodal cues. Results showed that the validity effect was found in AV and A cues, but no validity effect in unimodal V cues (Santangelo et al., 2006). There are other possibilities for the lack of Posner’s effect: we used headphones to present auditory signals, and this may affect the facilitation effect (Spence and Driver, 1997); Task requirements were different: we used the azimuth task (left vs. right), while other studies used the elevation task (up vs. down).

The limitation of the present study is that the experiments were performed in a laboratory environment, and the real flying experiment, which is necessary to avoid risks for the participants, was not performed. Also, the warning signals of the visual and auditory senses were mainly discussed, whereas other warning modalities (e.g., tactile sense) were not examined. In addition, the effect sizes in our study are relatively small. Combined with the results, although the bimodal warning signal has obvious advantages, the cockpit warning signal in actual flight should adopt a more rigorous grading system, and the integrated warning signal should be used in more dangerous situations. In some common signal designs, the use of unimodal warnings, especially audible warnings, can sufficiently elicit responses of the pilots. Moreover, according to “inattentional blindness,” people are likely to have insufficient visual/auditory stimuli awareness if stressing a distinct stimulus (Macdonald and Lavie, 2011; Murphy and Greene, 2017). Since flying commonly covers a significant visual perceptual load, the ability of the pilots to perceive the auditory warning signal may have an adverse effect. The combination of different senses should be used cautiously.

## Conclusion

In summary, temporal parameters were deployed to build integrated audiovisual warning signals. No matter in the case of low or high visual load, AV warning cues can effectively and efficiently arouse the attention of pilots. Specifically, A, AV, and VA100 warning cues increased spatial orientation to valid target stimuli in low load condition. With the increase in visual perceptual load, AV100, and A warning cues had a stronger spatial orientation to valid target stimuli. The results are expected to theoretically support the optimization design of the cockpit display interface, contributing to immediate flight crew awareness.

## Data Availability Statement

The original contributions presented in the study are included in the article/supplementary material, further inquiries can be directed to the corresponding author.

## Ethics Statement

The studies involving human participants were reviewed and approved by the Ethics Committee at the Civil Aviation Flight University of China. The patients/participants provided their written informed consent to participate in this study.

## Author Contributions

XP conceived the study, designed the experiments, and wrote the main manuscript text. HJ and RS performed the testing and data collection. JF and YL performed the statistical analysis and interpretation under the supervision of XP. JY reviewed the manuscript. All authors contributed to the manuscript revision, read, and approved the final version of the manuscript for submission.

## Conflict of Interest

YL was employed by the company Air China Limited. JF was employed by the company Beijing Capital Airlines. The remaining authors declare that the research was conducted in the absence of any commercial or financial relationships that could be construed as a potential conflict of interest.

## Publisher’s Note

All claims expressed in this article are solely those of the authors and do not necessarily represent those of their affiliated organizations, or those of the publisher, the editors and the reviewers. Any product that may be evaluated in this article, or claim that may be made by its manufacturer, is not guaranteed or endorsed by the publisher.
